# Magnetic Memory Effects in BaFe_2_(As_0.68_P_0.32_)_2_ Superconducting Single Crystal

**DOI:** 10.3390/ma17215340

**Published:** 2024-10-31

**Authors:** Alina M. Badea (Ionescu), Ion Ivan, Corneliu F. Miclea, Daniel N. Crisan, Armando Galluzzi, Massimiliano Polichetti, Adrian Crisan

**Affiliations:** 1 National Institute of Materials Physics, 405A Atomistilor Str., 077125 Magurele, Romania; alina.ionescu@infim.ro (A.M.B.); ion.ivan@infim.ro (I.I.); miclea@infim.ro (C.F.M.); daniel.crisan@infim.ro (D.N.C.); 2 Department of Physics “E.R. Caianiello”, University of Salerno, Via Giovanni Paolo II, 132, 84084 Fisciano, Italy; agalluzzi@unisa.it (A.G.); mpolichetti@unisa.it (M.P.)

**Keywords:** iron-based superconductors, anomalous peak effect, multi-harmonic susceptibility, magnetic memory effect, rhombic-to-square structural transition

## Abstract

Among many iron-based superconductors, isovalently substituted BaFe_2_(As_1−x_P_x_)_2_ displays, for *x* ≈ 0.3, apart from the quite usual Second Magnetization Peak (SMP) in the field dependence of the critical current density, an unusual peak effect in the temperature dependence of the critical current density in the constant field, which is related to the rhombic-to-square (RST) structural transition of the Bragg vortex glass (BVG). By using multi-harmonic AC susceptibility investigations in three different cooling regimes—field cooling, zero-field cooling, and field cooling with measurements during warming up—we have discovered the existence of a temperature region in which there is a pronounced magnetic memory effect, which we attributed to the direction of the structural transition. The observed huge differences in the third harmonic susceptibility at low and high AC frequencies indicates the difference in the time-scale of the structural transition in comparison with the timescale of the vortex excitations. Our findings show that the RST influence on the vortex dynamics goes beyond the previously observed influence on the onset of the SMP.

## 1. Introduction

The discovery of iron-based superconductors (IBSs) La(O_1−x_Fe_x_)FeAs, (x = 0.05–0.12) [1] with a critical temperature *T*_c_ = 26 K, with unique superconducting mechanism and potential of use in various applications led to a world-wide effort in the discovery and comprehensive studies of this new class of superconducting materials. Currently, there are literally thousands of possible chemical compositions and doping levels of various IBSs, of different types of crystalline structure. Among those different types of IBSs reported in the literature, 1111-type *RE*FeAsO_1−x_ (*RE* being a rare-earth element) has the highest critical temperature of 55 K [2]. Another type, namely 11-type Fe(Se,Te), has relatively low *T*_c_ (10–20 K depending on composition), but is promising as a thin film, having a high critical current density *J*_c_ in very high magnetic field (over 10^4^ A/cm^2^ in fields larger than 10 T) [3]. Lately, superconductors based on *AE*Fe_2_As_2_ (*AE* being alkali-earth metals Ca, Sr, Ba) parent compound, the so-called 122 system, became the most popular materials for both physical explorations and wire applications because of their *T*_c_ as high as 38 K [4], very high upper critical fields µ_0_*H*_c2_ (>70 T) [5,6] and low anisotropies γ (<2) [6], attracting substantial attention in comparison with other IBSs that have been reported in the literature. This is mainly due to the fact that in the 122 system, relatively large single crystals were relatively easy to grow using the self-flux technique [7], thus allowing more definitive characterization of the properties. The parent 122 compounds exhibit coupled structural and commensurate antiferromagnetic transitions, also called “spin density wave” transitions at around 100–200 K, resulting in distinct changes in the magnetic, thermal, and electrical properties. Superconductivity in *AE*Fe_2_As_2_ is primarily induced by alkali metal (*A* = Na, K, Rb, Cs) substitution at *AE* sites, with a concomitant suppression or elimination of the structural and magnetic ordering transition. However, this type of charge doping creates strong scattering potentials that affect superconducting properties such as the vortex pinning [8], the upper critical field [9], and even the superconducting gap symmetry [8]. Another type of 122 materials are isovalently substituted compounds such as Ba(Fe_1−x_Ru_x_)_2_As_2_ and BaFe_2_(As_1−x_P_x_)_2_, which are closer to the clean limit [10]. Partially replacing isoelectronic P for As in BaFe_2_As_2_ suppresses the long-range structural and antiferromagnetic transitions and induces superconductivity. In addition, unlike the vast majority of IBs, in BaFe_2_As_0.67_P_0.33_ crystals, penetration depth and thermal conductivity indicate the presence of line nodes in the multi-band superconducting order parameter [11], as well as an anomalous peak of the London penetration depth *λ*(0) for P doping close to the optimal one, most likely due to the vicinity of a quantum critical point [12]. In the same type of single crystals, slightly over-doped BaFe_2_As_0.68_P_0.32_, an anomalous peak in the isofield temperature dependence of magnetization (*m*(*T*)) was observed, suggesting a possible phase transition in the irreversible regime [13]. Although this system is in the clean limit, a vortex-glass phase was detected from isofield magneto-resistivity and multiharmonic AC susceptibility, and the second magnetization peak (SMP) was explained by the softening of the vortex lattice [14]. The possibility that the rhombic-to-square transition (RST) of the quasi-ordered vortex solid (the Bragg vortex glass, BVG) was responsible for the SMP was further investigated [15], but it was concluded that RST is not responsible for the SMP; instead, it can affect the onset of SMP if this is close to the (intrinsic) RST line, through the occurrence of a “shoulder” on the magnetic hysteresis curves *m*(*H*) and a maximum in the temperature variation of the DC critical current density. The *m*(*H*) shoulder is associated with a precipitous pinning-induced proliferation of dislocations at the RST, where the BVG elastic “squash” modulus softens. The DC magnetization relaxation indicates that the pinning-induced vortex system disordering continues above the RST domain, as the basic SMP mechanism. In this paper, we employ multi-harmonic susceptibility measurements in the three cooling-field protocols (zero-field cooling (ZFC), field cooling (FC), and field cooling–warming (FCW)) to further study the influence of the RST, weak pinning, and excitations timeframe on the vortex dynamics in BaFe_2_As_0.68_P_0.32_.

## 2. Materials and Methods

The BaFe_2_(As_0.68_P_0.32_)_2_ single crystals analyzed in this work were grown by the Ba_2_As_3_/Ba_2_P_3_-flux method [7] at the Institute of Physics, Chinese Academy of Sciences. They are square-shaped, with dimensions *a* ≈ 1.7 mm, *b* ≈ 1.7 mm and *c* ≈ 0.05 mm. Depending on the growth conditions, the amount of P in the BaFe_2_(As_1−x_P_x_) single crystals can be between *x* = 0.21 (heavily under-doped) and *x* = 0.64 (heavily over-doped). It was shown that the optimum doping, resulting in the largest critical temperature of 29 K, is with P content corresponding to *x* = 0.3. The BaFe_2_(As_0.68_P_0.32_)_2_ sample studied here is a slightly over-doped sample with a critical temperature of 27.8 K.

The multi-harmonic AC susceptibility studies were performed using a commercial Quantum Design Inc (San Diego, CA, USA) Physical Property Measurement System (PPMS) using the ACMS option, which allows up to 10 kHz and AC field amplitudes up to 16 Oe, and up to 14 T in DC fields. It is well established that the third harmonic signal offers much more information on nonlinear vortex dynamics [16]. Isothermal magnetization loops were also performed with PPMS, using the VSM option. It should be noted that due to a small problem of an electronic component, DC fields with positive (up) orientation could be achieved in a controllable way only up to 8 T, while in the opposite direction, we could obtain the maximum 14 T; hence, the incomplete isothermal magnetization loops. In all of the experiments reported here, the applied field (both DC and AC) is parallel to the *c*-axis (perpendicular to the largest plane *a*–*b*) of the single crystals.

## 3. Results

After confirmation of the quality of the single crystal by a very basic temperature dependence of susceptibility we proceeded with the measurement of the isothermal hysteresis magnetization loops using the VSM option, and of the temperature dependence of multi-frequency AC susceptibility in the three cooling conditions, ZFC, FC, and FCW.

### 3.1. Isothermal Magnetization Loops and Critical Current Density

**Figure 1:**
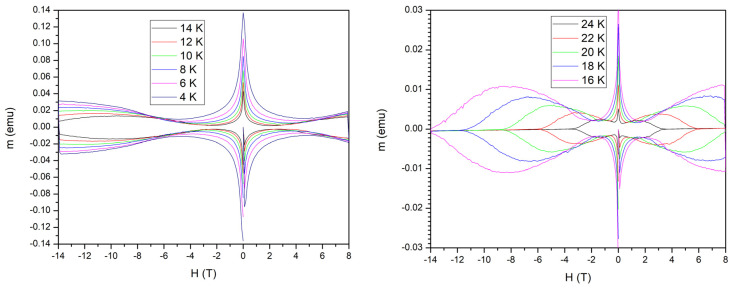
Isothermal magnetization loops at the temperatures indicated in the figure.

Figure 1 shows the results of the isothermal magnetic field dependence of the magnetic moment, *m*(*H*). For clarity, we have separated the results in two panels, for temperatures between 4 and 14 K (2 K step) in the left-hand-side panel, and, respectively, between 16 and 24 K (2 K step) in the right-hand-side panel.

Clearly visible is a very pronounced second magnetization peak (SMP), also called, in some literature, the “fish-tail” effect, which is a nonmonotonic variation of Δ*m*(*H*) = *m*(*H*)↓ − *m*(*H*)↑, with *m*(*H*)↑ being the magnetization in an increasing magnetic field (in module), and *m*(*H*)↓ being the magnetization measured in a decreasing magnetic field. A closer inspection of the right-hand-side panel also reveals a very clear intersection of some isothermal magnetization loops, leading to the anomalous peak effect in isofield *m*(*T*) dependencies. Neglecting the low field data (for fields smaller than the lower critical field *H*_c1_), from the Δ*m*(*H*) data, we can estimate the critical current density as a function of temperature and field, using the modified Bean critical state model [17]. With the dimensions of the rectangle (the face of the sample perpendicular to the magnetic field) *l* (length) and *w* (width), with *l* > *w*, and the sample dimension in the field direction *d* (thickness), the critical current density, in A/cm^2^, is given by

(1) $$J_{c}=\frac{60\Delta m}{w^{2}d\left(3l-w\right)}\;,$$

with Δ*m*(*H*) = *m*(*H*)↓ − *m*(*H*)↑ measured in emu, and all three dimensions in cm. If the sample is a square plate, i.e., *l* = *w* (which is the case of our sample), Equation (1) becomes

(2) $$J_{c}=\frac{30\Delta m}{w^{3}d}$$

**Figure 2:**
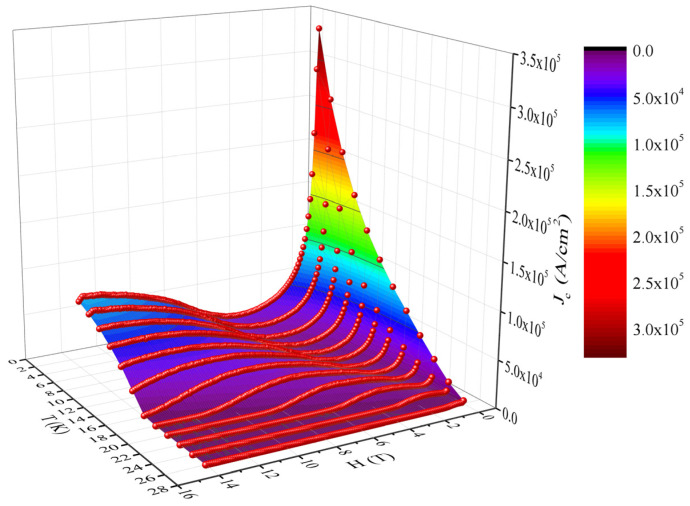
Three-dimensional graph of the temperature and DC field dependence of the critical current density obtained from the magnetization hysteresis loops in Figure 1, using the modified critical state Bean model.

From the experimental results of the measurements of isothermal magnetization loops shown in Figure 1, we evaluated the values of the critical current density as a function of field and temperature *J*_c_(*H*, *T*), as shown in the three-dimensional plot in Figure 2.

As can be seen, the values of the critical current density are relatively modest. “Slices” in the 3D surface for *T* = *const*. show the fish-tail effect (SMP) in *J*_c_(*H*); also there are regions in which “slices” in the 3D surface for constant *H* show the anomalous *J*_c_(*T*) peak.

### 3.2. Multiharmonic AC Susceptibility in H_DC_ = 1 T

**Figure 3:**
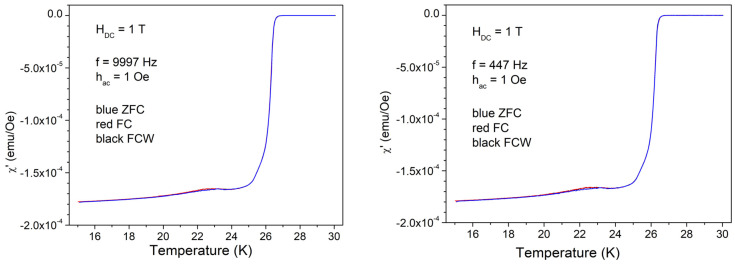
Temperature dependence of the in-phase susceptibility in DC field of 1 T, amplitude of AC field of 1 Oe, and frequencies of AC field of 447 and, respectively, 9997 Hz, cooling regimes indicated by the different colors.

For a systematic study, we have chosen, for each DC applied field, AC field excitations with one low frequency of 447 Hz, one high frequency of 9997 Hz, one low amplitude of excitation (1 Oe), and one high amplitude of 10 Oe, in the three cooling regimes (ZFC, FC, and FCW), i.e., a total of 12 complex multi-harmonic susceptibility curves for each DC field. In the following, we will show the in-phase and out-of-phase components of the fundamental and third harmonic susceptibility, neglecting the second harmonic and higher harmonics. Figure 3 shows the temperature dependence of in-phase fundamental susceptibility with low *h*_AC_.

It can be seen that the diamagnetic signal is a usual one, quite sharp, with a small anomaly around 22–23 K. There are very small, practically indistinguishable differences between the three cooling protocols, and there are no visible differences between the low and high excitation frequencies.

**Figure 4:**
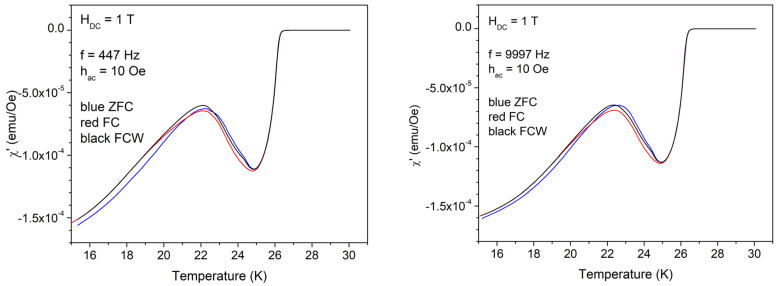
Temperature dependence of the in-phase susceptibility in a DC field of 1 T, the amplitude of the AC field of 10 Oe, and frequencies of the AC field of 447 and, respectively, 9997 Hz; cooling regimes indicated by the different colors.

In the case of high amplitude of excitation *h*_AC_ = 10 Oe, the results are completely different, as can be seen in Figure 4. After the usual increase in the diamagnetic signal, at around 25 K, there is a very abnormal decrease in the diamagnetic signal (decrease in the shielding supercurrent), and, below 22 K, the trend returns to the normal behavior of increasing the diamagnetic signal with decreasing temperature. A closer look reveals that at high temperatures, there is no difference between ZFC, FC, and FCW; in the intermediate temperature range where the anomaly is present, ZFC, FC, and FCW signals are different, while at low temperatures, FC and FCW are the same, with the ZFC signal being different, as expected for usual superconductors in the irreversible regime. Apparently, there are no differences between the low and high frequencies; however, a closer look shows that FC and FCW start to be the same at about 19 K at low frequency, and at 20 K at high frequency.

**Figure 5:**
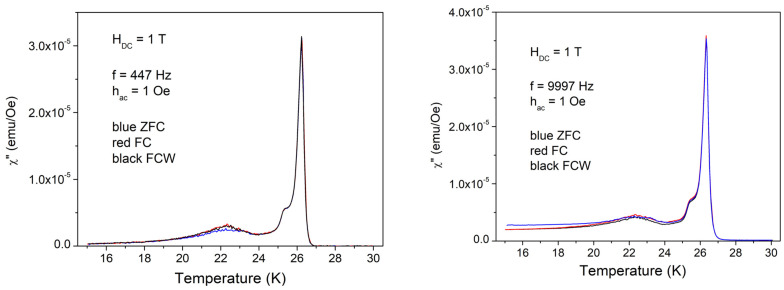
Temperature dependence of the out-of-phase susceptibility in a DC field of 1 T, the amplitude of the AC field of 1 Oe, and frequencies of the AC field of 447 and, respectively, 9997 Hz; cooling regimes indicated by the different colors.

**Figure 6:**
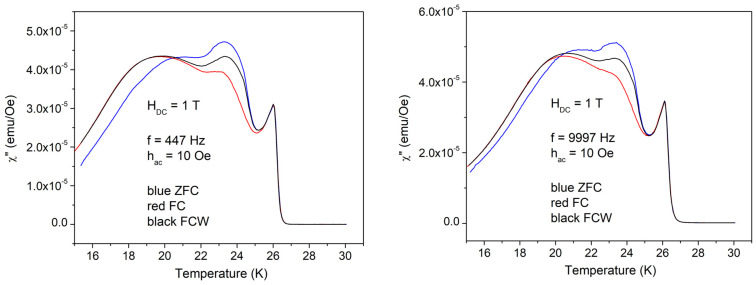
Temperature dependence of the out-of-phase susceptibility in a DC field of 1 T, the amplitude of AC field of 10 Oe, and frequencies of the AC field of 447 and, respectively, 9997 Hz; cooling regimes indicated by the different colors.

Figure 5 and Figure 6 show the temperature dependence of the out-of-phase susceptibility.

**Figure 7:**
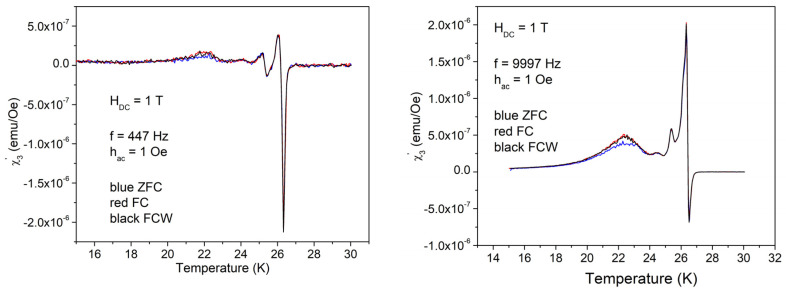
Temperature dependence of the in-phase third harmonic susceptibility in DC field of 1 T, amplitude of the AC field of 1 Oe, and frequencies of the AC field of 447 and, respectively, 9997 Hz; cooling regimes indicated by the different colors.

Also, in this case, there are huge differences between low and high amplitudes of the AC field excitations. With 1 Oe, there is the usual dissipation peak with some shoulder and a very small local maximum at 22–23 K. High frequency also results in a small difference between ZFC and FC = FCW cooling protocols. With the high amplitude of the excitation field of 10 Oe, the dissipation in a wide range of temperatures is much higher than the sharp dissipation peak at *T*_c_, with very peculiar features, and with three distinct regions from the point of view of magnetic memory: (i) high temperatures where ZFC = FC = FCW, reversibility region; (ii) intermediate temperatures where ZFC ≠ FC ≠ FCW, irreversibility and magnetic memory region; and (iii) low temperatures where ZFC ≠ FC = FCW, irreversibility without magnetic memory region. The value of frequency doesn’t change significantly the χ″(*T*) curves. Figure 7 shows the temperature dependence of the in-phase third harmonic susceptibility for the AC field amplitude of 1 Oe. Differences between ZFC, FC, and FCW are very small, again around 22 K, but the differences due to different frequencies are significant. At low frequency, the highest peak is just after the superconducting transition and has “negative” value, while at higher frequency, the first peak, negative, is not the highest one, with the main peak and the other smaller features being positive.

**Figure 8:**
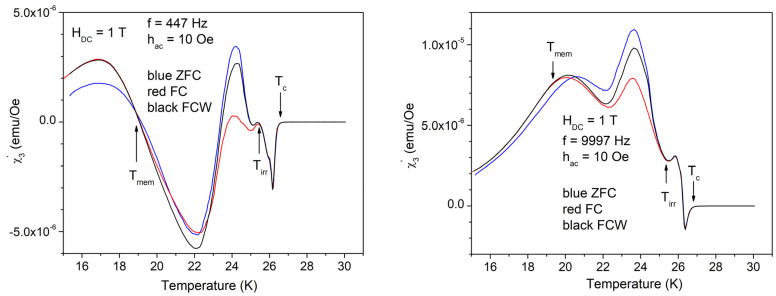
Temperature dependence of the in-phase third harmonic susceptibility in the DC field of 1 T, the amplitude of the AC field of 10 Oe, and frequencies of the AC field of 447 and, respectively, 9997 Hz; cooling regimes indicated by the different colors.

Temperature dependence of the in-phase third harmonic is much more spectacular with high amplitude of the AC field of 10 Oe, as can be seen in Figure 8.

As can be seen in Figure 8, the in-phase third harmonic susceptibility shows completely different features for low and, respectively, high frequency, which is not the case of the fundamental susceptibility shown in Figure 4 and Figure 6, where the differences due to different frequencies are insignificant. Arrows in Figure 8 indicate, from high to low temperature: *T*_c_, the temperature of superconducting transition (critical temperature); *T*_irr_, the temperature at which the irreversibility and magnetic memory region starts; and, respectively, *T*_mem_, the temperature at which the magnetic memory effect ends, but the irreversibility region continues towards lower temperatures. In other words, *T* < *T*_mem_ is the region of irreversible regime without memory effect (ZFC ≠ FC = FCW), *T*_mem_ < *T* < *T*_irr_ is the region of irreversibility and magnetic memory effect (ZFC ≠ FC ≠ FCW), *T*_irr_ < *T* < *T*_c_ is the reversibility region, and *T* > *T*_c_, is the non-superconducting (normal) region.

**Figure 9:**
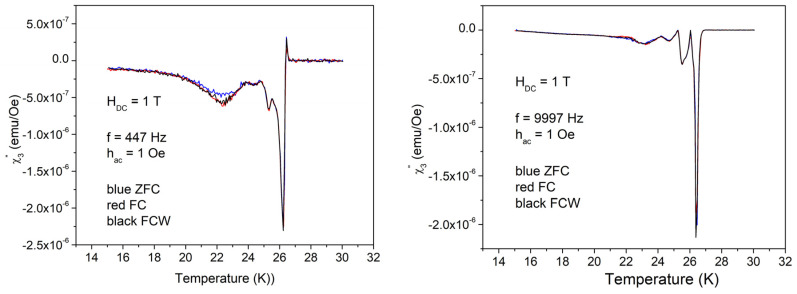
Temperature dependence of the out-of-phase third harmonic susceptibility in the DC field of 1 T, the amplitude of the AC field of 1 Oe, and frequencies of the AC field of 447 and, respectively, 9997 Hz.

The out-of-phase third harmonic susceptibility measured with low AC field of 1 Oe, shown in Figure 9, also presents differences for the two frequencies, but not clear separation of different regions.

**Figure 10:**
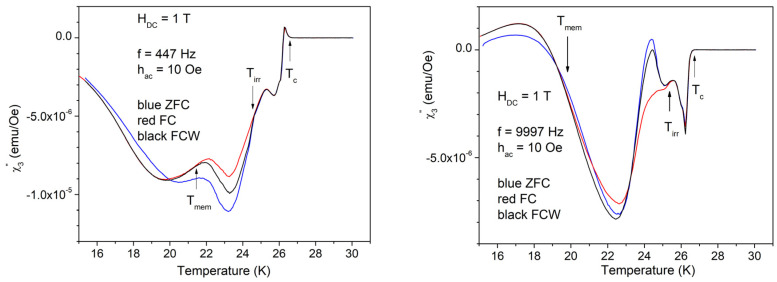
Temperature dependence of the out-of-phase third harmonic susceptibility in the DC field of 1 T, the amplitude of the AC field of 10 Oe, and frequencies of the AC field of 447 and 9997 Hz; cooling regimes indicated by the different colors.

With the high AC field amplitude of 10 Oe, the out-of-phase AC susceptibility shows very different features at the low and high frequencies, as well as very clear delimitation of the regions with different vortex dynamics and magnetic memory effects, as can be seen in Figure 10. Arrows indicate the same temperatures as discussed for the in-phase third harmonic susceptibility.

After the first set of measurements in the DC field of 1 T, we could see several important facts. A small AC field amplitude is too small to influence significantly, deep into the sample, the supercurrent profile of the critical state induced by the DC field. Relevant information regarding vortex dynamics and the magnetic memory effect appears only for a higher AC excitation field of 10 Oe, when the perturbations of the critical state induced by the DC field reaches the whole sample. The fundamental susceptibility, both in-phase and out-of-phase, shows very small difference for small or high AC field frequencies. The third harmonics, however, have completely different features for low and high frequencies. The temperatures described in the text and indicated by arrows in some of the figures seem to be the same in the fundamental and third harmonics, regardless of frequency, with small variations within experimental errors and our choice of looking at the three curves. For this reason, from the measurements done at higher fields, we will show just a part of the results, all of the data being available on request.

### 3.3. Multiharmonic AC Susceptibility in Higher DC Fields

**Figure 11:**
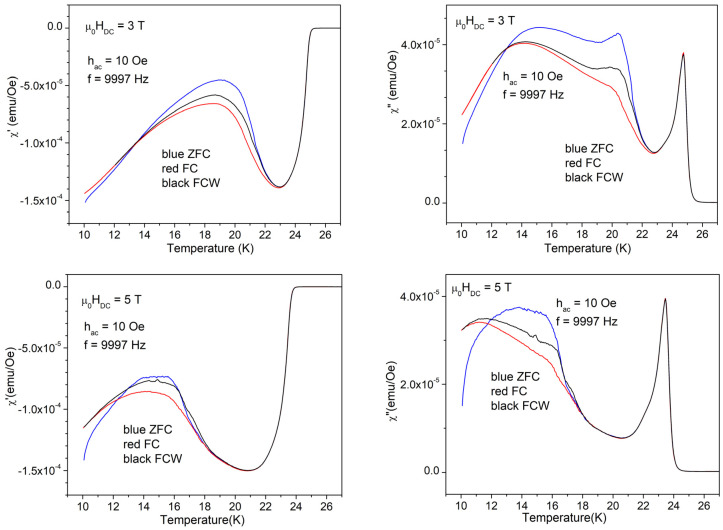

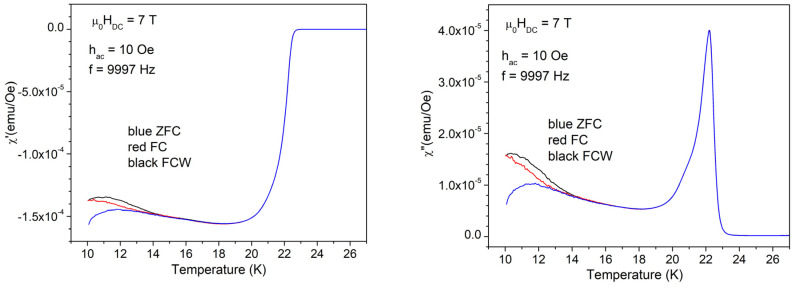
Temperature dependence of the in-phase (left-hand-side) and out-of-phase (right-hand-side) fundamental susceptibility measured with the amplitude of AC field of 10 Oe, and the frequency of AC field of 9997 Hz, in the fields (top to bottom) of 3, 5, and 7 T; cooling regimes indicated by the different colors.

**Figure 12:**
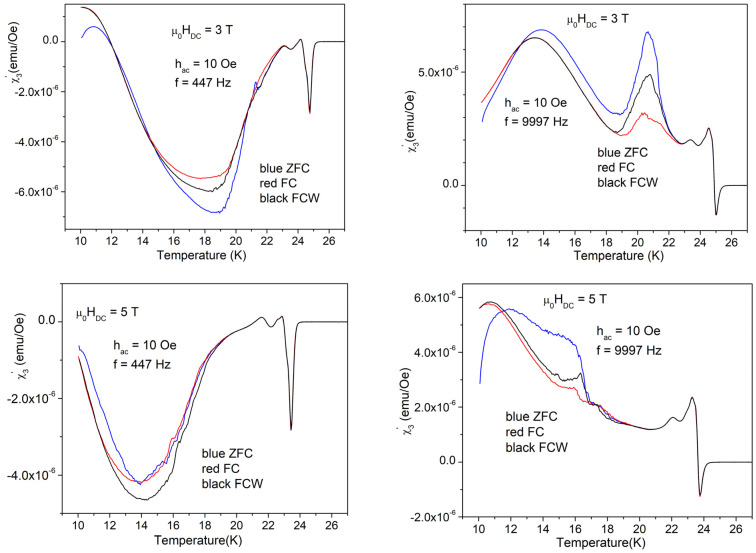

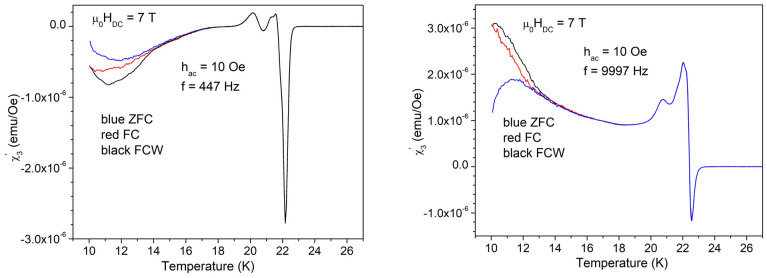
Temperature dependence of the in-phase third harmonic susceptibility measured with the amplitude of AC field of 10 Oe and the low frequency of 447 Hz (left-hand-side) and, respectively, with the high frequency of 9997 Hz (right-hand-side) in the fields (top to bottom) of 3, 5, and 7 T; cooling regimes indicated by the different colors.

**Figure 13:**
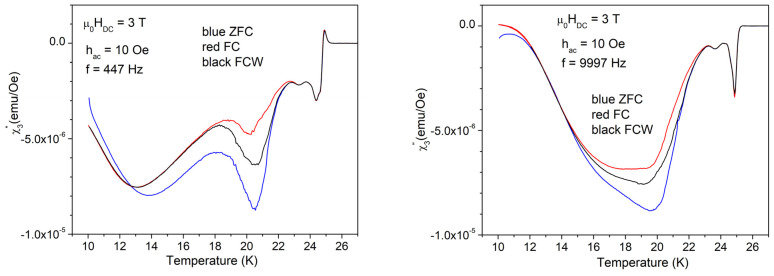

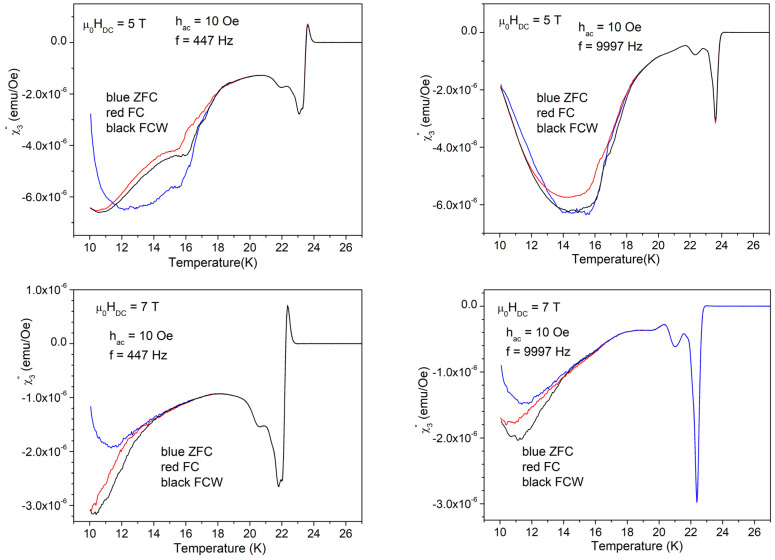
Temperature dependence of the out-of-phase third harmonic susceptibility measured with the amplitude of AC field of 10 Oe and the low frequency of 447 Hz (left-hand-side) and, respectively, with the high requency of 9997 Hz (right-hand-side) in the fields (top to bottom) of 3, 5, and 7 T, cooling regimes indicated by the different colors.

As mentioned before, from the data obtained in higher fields, we will present only those with an AC field amplitude of 10 Oe, and, for fundamental susceptibility, for just one frequency. For a clear view of the changes induced by different DC fields, we will present the results of various susceptibility components in a column, for the three DC fields of 3, 5, and 7 T. Figure 11 shows the in-phase (left-hand-side) and out-of-phase (right-hand-side) fundamental susceptibility in the three DC fields, measured with a DC field amplitude of 10 Oe and frequency of 9997 Hz. It can be seen that, with increasing DC field, all three temperatures of interest—*T*_c_ (temperature of superconducting transition), *T*_irr_ (temperature of irreversible magnetization), and *T*_mem_ (temperature where the magnetic memory disappears)—move to lower temperatures. Figure 12 and Figure 13 present the in-phase third harmonic susceptibility, and, respectively, out-of-phase third harmonic susceptibility, for a low AC frequency of 447 Hz (left-hand-side), and a high frequency of 9997 Hz, for the three DC fields.

It can be seen that, similarly to the fundamental susceptibility, for both in-phase and out-of-phase third harmonic susceptibility, the three relevant temperatures that separate various regimes of vortex dynamics move to lower temperatures with increasing DC fields. It is worth noting that the general features which are totally different for the two frequencies have the same trends in different DC fields, even with distortions and translations to lower temperatures, a clear evidence of the huge importance of the timescale for vortex dynamics in this material.

## 4. Discussion

The DC magnetization loops and the field and temperature dependence of the resulting critical current density calculated using the modified critical state model showed that the single crystal investigated in this work is qualitatively the same (has the same superconducting properties) as in the case of single crystals with the same compositions used in Refs. [13,14,15]. In brief, in Ref. [13] were observed for the first time, in a single crystal with the same composition, intersections of isothermal magnetization loops, very similar to what we obtained and presented in Figure 1, as well as an anomalous peak in the temperature dependence of magnetization at certain fixed DC fields, which translates into an anomalous peak in the temperature dependence of the critical current density, which is also visible for our sample upon a closer look at Figure 2 (imaginary slices in the *J*_c_(*T*,*H*_DC_) surface along several *H*_DC_ = *const* planes). On similar single crystals, the onset of third harmonic susceptibility was employed to probe the vortex liquid–vortex glass transition [14]; in our multi-harmonic susceptibility measurements, such onset, at the same DC field, is situated at the same temperature. In a study of the second magnetization peak and rhombic-to-square Bragg vortex glass transition [15] on a single crystal from the same batch as the one used for our paper, the shapes of the magnetization loops and several intersections of such curves were also observed, and they are similar to the results shown here.

By far, the most striking properties of this material that resulted from our multi-harmonic, multi-frequency AC susceptibility studies are (i) the existence of a temperature region in which, after the superconducting transition occurs, the diamagnetic screening (i.e., the screening current density) decreases with decreasing temperature; (ii) the existence of a temperature region in which the susceptibility response of the sample is different for the case of field-cooling experiments for decreasing and, respectively, increasing the temperature (magnetic memory effect); and (iii) the huge difference in the third harmonic susceptibility response for low and, respectively, high frequency of the AC field excitation.

As mentioned before, the three characteristic temperatures, *T*_mem_ < *T*_irr_ < *T*_c_, and the temperature region of magnetic memory effect, *T*_mem_ < *T* < *T*_irr_, move to lower temperatures with increasing DC field. It can be seen from the measurements shown in Figure 4 and Figure 11 (left hand side) that the anomaly in the diamagnetic screening (the anomalous suppression of the screening current density—the “peak” in the χ’(*T*))— is larger at *H*_DC_ = 3 T. In this DC field, a careful look at the width of the magnetic memory region, Δ*T* = *T*_irr_ − *T*_mem_, shows a striking feature: for the in-phase third harmonic susceptibility, Δ*T* = 23 K − 18 K at 9997 Hz is much smaller than Δ*T* = 23 K − 14 K at 447 Hz. More puzzling, upon looking at the out-of-phase third harmonic susceptibility, the situation is reversed, Δ*T* being smaller at the low frequency. Not only from the completely different shapes of the harmonic response at different frequency, but also from this frequency-dependent width of the magnetic memory region, it is obvious that the vortex system and its dynamics strongly depend on the frequency of the AC field excitation.

Since this crystal is characterized by weak pinning centers, as shown by the small values of the critical current density, the vortex system is, in fact, a quasi-ordered Bragg vortex glass (BVG). In addition, since the crystal structure has a four-fold symmetry, the BVG can undergo a rhombic-to-square (RST) phase transition [13,14]. The second magnetization peak (SMP) was explained by the pinning-induced disordering of the BVG, while the RST has an influence on the onset of the SMP [15].

From our susceptibility studies, it appears that the rhombic-to-square phase transition of the Bragg vortex glass has much wider implications on the vortex dynamics in this system. In FC and FCW experiments, the trapped DC magnetic field is the same, so the existence of the magnetic memory effect (different results when cooling and warming) can, in our opinion, arise only due to the RST, transformation from rhombic to square in increasing *T*, or from square to rhombic in decreasing *T*. The RST occurs gradually, in a temperature range of several K, at temperatures that are field-dependent. The timescale of the structural transition depends on the rate of cooling or heating during the measurements, but is most likely different than that of the timescale of vortex dynamics, so the temperature width of the magnetic memory effect, as well as the shapes of the harmonic susceptibility components, are strongly frequency-dependent.

## 5. Conclusions

In conclusion, through DC magnetization hysteresis loops, we confirmed that the investigated BaFe_2_(As_0.68_P_0.32_)_2_ single crystal has a very pronounced second magnetization peak in the *M*(*H*) dependence at constant temperatures as well as an anomalous peak effect in the *M*(*T*) dependence at fixed fields, with an overall quite small critical current density, indicating a very weak pinning. Due to this weak pinning, the vortex system in this sample is a Bragg vortex glass which, in the case of four-fold symmetry, can undergo a rhombic-to-square structural phase transition. Multi-harmonic AC susceptibility studies performed in the three cooling/field protocols ZFC, FC, and FCW revealed the existence of three different regions in temperature regarding vortex dynamics: the reversibility region at high temperatures, the irreversibility region with magnetic memory effect at intermediate temperatures, and the irreversibility region without magnetic memory effect at low temperatures. We have attributed the appearance of the magnetic memory effect to the influence of the rhombic-to-square structural transition on the vortex dynamics, with an additional proof being the strong dependence of the third harmonic susceptibility on the AC frequency.

## Data Availability

The original contributions presented in the study are included in the article, further inquiries can be directed to the corresponding author.
